# Emergent neurovascular imaging in patients with blunt traumatic injuries

**DOI:** 10.3389/fradi.2022.1001114

**Published:** 2022-09-15

**Authors:** Michael T. Bounajem, J. Scott McNally, Cordell Baker, Samantha Colby, Ramesh Grandhi

**Affiliations:** ^1^ Department of Neurosurgery, Clinical Neurosciences Center, University of Utah, Salt Lake City, UT, United States; ^2^ Department of Radiology, University of Utah, Salt Lake City, UT, United States

**Keywords:** blunt cerebrovascular injury, imaging, screening, computed tomography angiography, digital subtraction angiography, magnetic resonance imaging, transcranial doppler, review

## Abstract

Blunt cerebrovascular injuries (BCVIs) are commonly encountered after blunt trauma. Given the increased risk of stroke incurred after BCVI, it is crucial that they are promptly identified, characterized, and treated appropriately. Current screening practices generally consist of computed tomography angiography (CTA), with escalation to digital subtraction angiography for higher-grade injuries. Although it is quick, cost-effective, and readily available, CTA suffers from poor sensitivity and positive predictive value. A review of the current literature was conducted to examine the current state of emergent imaging for BCVI. After excluding reviews, irrelevant articles, and articles exclusively available in non-English languages, 36 articles were reviewed and included in the analysis. In general, as CTA technology has advanced, so too has detection of BCVI. Magnetic resonance imaging (MRI) with sequences such as vessel wall imaging, double-inversion recovery with black blood imaging, and magnetization prepared rapid acquisition echo have notably improved the utility for MRI in characterizing BCVIs. Finally, transcranial Doppler with emboli detection has proven to be associated with strokes in anterior circulation injuries, further allowing for the identification of high-risk lesions. Overall, imaging for BCVI has benefited from a tremendous amount of innovation, resulting in better detection and characterization of this pathology.

## Introduction

Blunt cerebrovascular injuries (BCVIs) include any injury to the cervical vasculature (carotid arteries, vertebral arteries, and their respective branches) that occurs in the setting of a blunt, non-penetrating, traumatic injury. BCVIs are found in up to 7% of all blunt traumatic injuries (1, 2), with the most common cause being high-velocity incidents such as motor vehicle accidents, falls (particularly from heights), and motor vehicle vs. pedestrian crashes (3). Although only present in a small percentage of blunt traumas, BCVIs are clinically significant given that they carry an increased risk for developing ischemic stroke, with some studies reporting post-BCVI stroke rates as high as 5%–28% (1, 3, 4). Diagnosis is incumbent on screening protocols, which determine the need for non-invasive imaging such as computed tomography angiography (CTA) and possibly more invasive procedures such as digital subtraction angiography (DSA). Although the implementation of CTA has led to improved diagnosis of BCVI and less delay in treatment (5), there is still significant debate regarding whether it is the optimal imaging study for identifying and further characterizing BCVIs. This issue is twofold in nature: first, CTA has demonstrated variable rates of sensitivity and positive predictive value in the identification of true BCVIs, and second, CTA may miss high-risk features of an injury such as the presence of microemboli (6, 7). The purpose of this review is to describe the current state of emergent neurovascular imaging techniques for the diagnosis, characterization, and management of vascular injuries in patients presenting with blunt traumatic injuries.

## Methods

An initial query of PubMed, Embase, and Scopus was conducted using the following search criteria: “screening” OR “imaging” AND “blunt cerebrovascular injury.” Subsequent queries were conducted using criteria including “magnetic resonance imaging” AND “blunt cerebrovascular injury,” “computed tomography” AND “blunt cerebrovascular injury,” and “transcranial doppler” AND “blunt cerebrovascular injury.” Original articles examining advances in imaging methods employed in the diagnosis and evaluation of BCVI were included. Exclusion criteria included review articles and publication in non-English languages.

## Results

**Figure 1:**
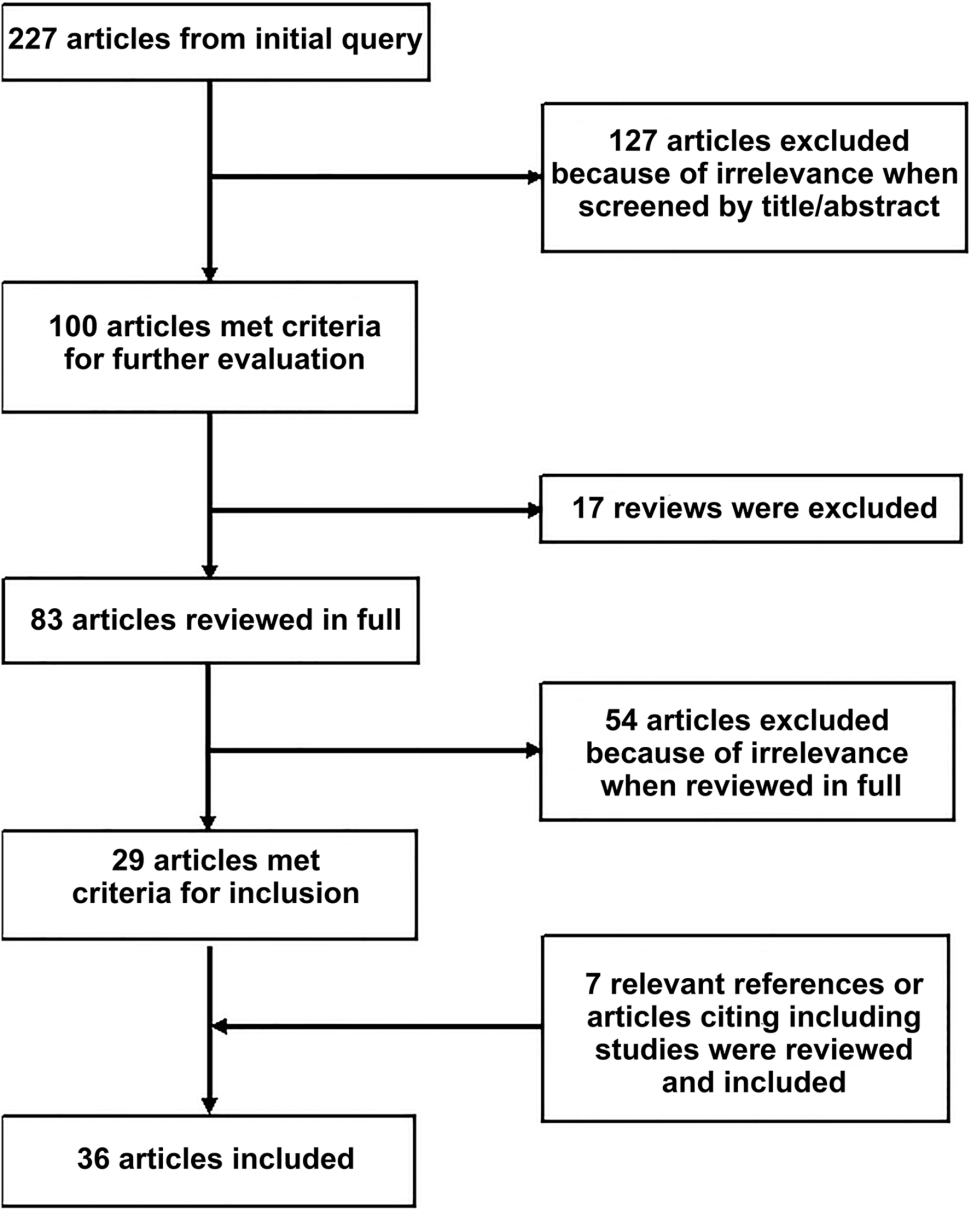
PRISMA flow chart illustrating article identification.

These queries resulted in 227 articles, which were then screened by title and abstract for relevance to the topic at hand and narrowed to a list of 100 articles. Reviews were assessed for any relevant missed primary articles but were not included in the final list of studies. References of included articles were also examined for relevant studies, as were articles citing the included works. Thirty-six articles were identified for inclusion in the final analysis (Figure 1). Articles directly examining the rate of BCVI detection or detection of high-risk features have been summarized in Table 1.

**Table 1:** Articles assessing accuracy of imaging modalities in detecting BCVIs and high-risk features.

Authors and year	Imaging modality	Modality subcategory	No. patients evaluated	Objective	Accuracy metric	Accuracy value
Be et al., 2004	CTA	Single detector	486	BCVI detection	PPV	37.5%
Berne et al., 2006	mdCTA	16-channel	435	BCVI detection	Incidence	5.5%
Eastman et al., 2006	mdCTA	16-channel	146	BCVI detection	Sensitivity	97.7%
					Specificity	100%
					PPV	100%
					NPV	99.3%
Malhotra et al., 2007	mdCTA	16-channel	119	BCVI detection	Sensitivity	74%
					Specificity	86%
					PPV	65%
					NPV	90%
Goodwin et al., 2009	mdCTA	16-channel	74	BCVI detection	Sensitivity	29%
		64-channel	84	BCVI detection	Sensitivity	54%
DiCocco et al., 2011	mdCTA	32-channel	684	BCVI detection	Sensitivity	51%
Paulus et al., 2014	mdCTA	64-channel	594	BCVI detection	Sensitivity	68%
					Specificity	92%
					PPV	36.2%
					NPV	97.5%
Kik et al., 2022	mdCTA	Varied	1,918 (meta-analysis)	BCVI detection	Sensitivity	64%
					Specificity	95%
Grandhi et al., 2018	mdCTA	64-channel	Not specified	BCVI detection	False positive	47.9%
					PPV (Gr I injuries)	30%
					PPV (Gr II injuries)	76%
					PPV (Gr IV injuries)	97%
Biffl et al., 2002	MRA		16	BCVI detection	Sensitivity	75%
					Specificity	67%
					PPV	43%
					NPV	89%
Adam et al., 2020	MRI	DWI	50	Intramural hematoma detection	Sensitivity	86%–90%
Mutze et al., 2005	TCD		1,471	BCVI detection	Sensitivity	38.5%
					Specificity	100%
Bonow et al., 2017	TCD	Emboli detection	1,146	Association with stroke when 1+ microembolus detected	Risk ratio	5.05

mdCTA, multidetector computed tomography angiography; MRA, magnetic resonance angiography; MRI, magnetic resonance imaging; DWI, diffusion-weighted imaging; TCD, transcranial doppler; PPV, positive predictive value; NPV, negative predictive value.

## Discussion

### CT angiography

Screening for blunt cerebrovascular injury begins at the level of clinical evaluation, with high-risk features including chest trauma, high-energy injury, neck and spine injuries, male sex, skull base fractures, hypotension, and low Glasgow Coma Scale score (GCS) (8, 9). Given the often complex nature and high acuity of these injuries, the identification of BCVIs, which do not necessarily demonstrate immediate signs or symptoms, can be easily neglected; guidelines therefore clearly indicate that clinical screening protocols are beneficial and lead to greater rates of BCVI detection (10). Significant research efforts have therefore been dedicated to the optimization and vetting of screening protocols such as the Denver Criteria and the Memphis Criteria. Although they each have their unique specifications, the Denver and Memphis criteria both endorse vascular imaging for patients who present with skull base fractures, cervical spine fractures, soft tissue injury to the neck, or focal neurological deficit in the setting of blunt traumatic injury (11); they may nonetheless miss some BCVIs. Black et al. (11) recently reported the experience at their Level I trauma center, where all patients with blunt traumatic injuries (excluding those with isolated extremity injuries and those in which the attending trauma surgeon deferred CTA) have undergone CTA since 2016. The authors retrospectively assessed the efficacy of their “universal screening” protocol and compared the results with traditional screening criteria *via* the Denver Criteria, extended Denver Criteria, and Memphis criteria. They found that 42.5%, 25.3%, and 52.7% of injuries, respectively, would have been missed had the patients received imaging strictly based on the screening criteria (11). Given the evidence for missing injuries using the traditional screening criteria and the consequent potential for increased strokes due to undiagnosed BCVI, many centers such as ours have adopted a policy that all patients that have sustained a high-velocity blunt trauma undergo a screening CTA of the neck, which includes the entire length of the carotid and vertebral arteries from their origin inferiorly up to the level of the Circle of Willis superiorly. This is conducted during initial imaging surveys by the trauma service in patients who are stable enough to undergo imaging or at a later time if patients have hemodynamic instability or other more acute needs that preclude the acquisition of imaging studies.

**Figure 2:**
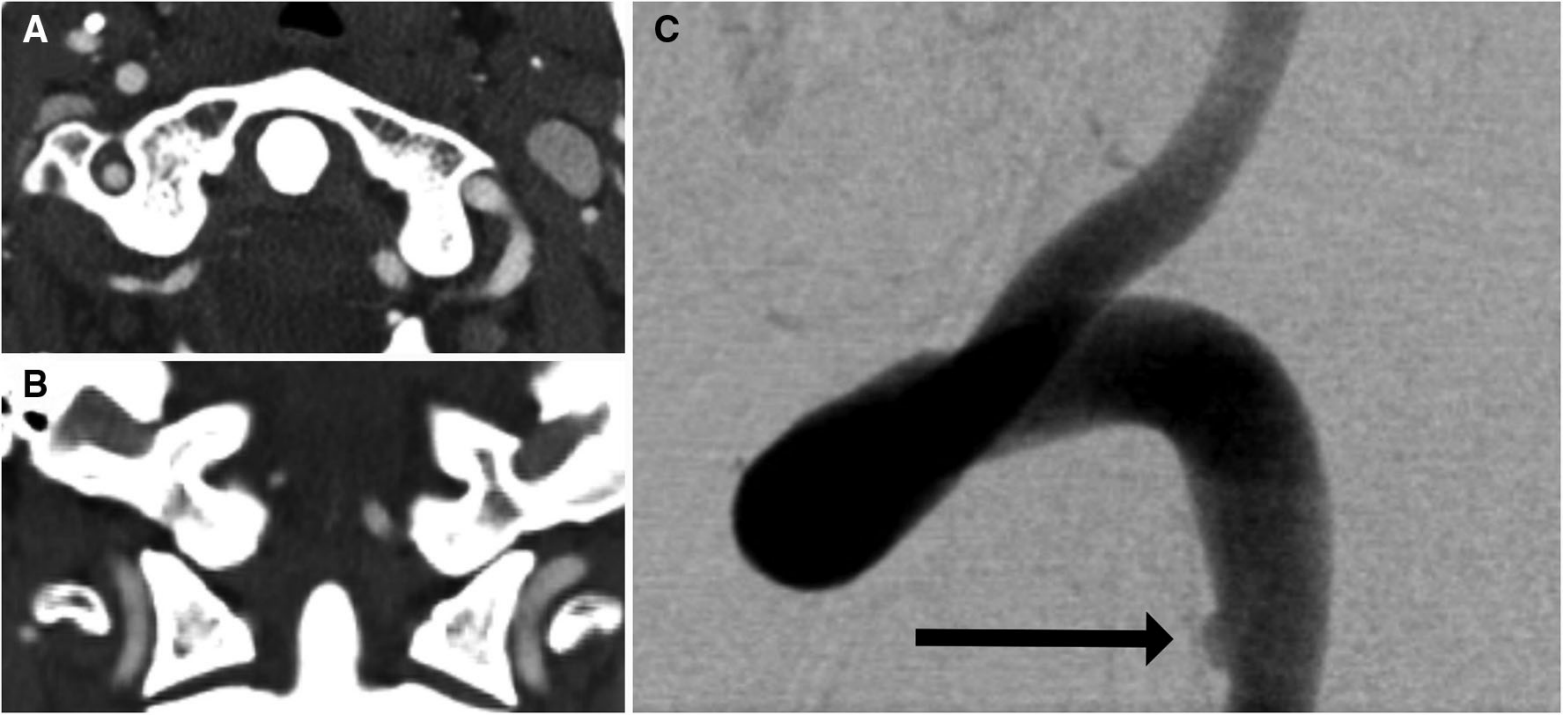
Imaging of a 35-year-old man who presented after a motor vehicle crash. Initial axial (**A**) and coronal (**B**) CTA were negative for vertebral artery dissection. Because of a high level of suspicion for vertebral artery injury, DSA (**C**) was performed. DSA showed subtle intimal injury involving the left vertebral artery (arrow).

**Figure 3:**
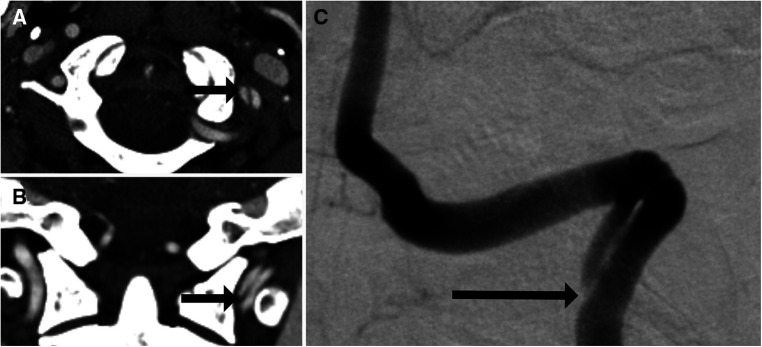
One-month follow-up imaging of the 35-year-old man shown in Figure 1. Axial (**A**) and coronal (**B**) imaging showed interval development of a left vertebral artery dissection flap (arrows). This was confirmed on DSA (**C**) with the dissection flap beginning at the site of prior intimal disruption (arrow).

For many years, catheter-based DSA had been the diagnostic tool of choice for BCVIs. Although it is still considered to be the gold standard given the superior detail it offers, the vast majority of patients with potential BCVI are now imaged *via* CTA, followed by DSA if needed. At its inception, CTA for BCVI screening consisted of single-detector helical acquisition, which yielded high levels of sensitivity in isolated smaller series of patients, but poor positive predictive value [e.g., 37.5% in Berne et al. (12)]. As CT technology improved, further assessments were conducted with multidetector sequences, starting with 4-, 16-, 32-, and 64-channel multidetector arrays, with some institutions now implementing 256-channel arrays as well. As the number of arrays increased, so too did incidence of BCVI detected. Berne et al. reported a rate of BCVIs among patients screened with 16-channel multidetector CTA (mdCTA) of 5.5%, compared with the previously reported 1.2% obtained with 4-channel mdCTA (13). Furthermore, the use of multidetector sequences potentially increased sensitivity. Eastman et al. (14) reported that CTA had a sensitivity of 97.7% in a series of 146 patients who underwent both 16-channel mdCTA and DSA. Malhotra et al. (15) reported a sensitivity in 16-channel mdCTA of 74%. These results were, however, not reliably replicable. For example, Goodwin et al. (7) compared mdCTA (a combination of 16- and 64-channel) with DSA in a series of 158 patients. Sixteen-channel mdCTA demonstrated a sensitivity of 29% (95% CI: 0.08–0.58), whereas 64-channel mdCTA demonstrated a sensitivity of 54% (95% CI: 0.25–0.81). DiCocco et al. (16) demonstrated a similar sensitivity (51%) for 32-channel mdCTA when compared with DSA. Further analyses of 64-channel mdCTA by Paulus et al. (17) demonstrated a sensitivity of 68%, which was a significant increase over prior reports. Despite improving reports, a recent meta-analysis conducted by Kik et al. (18) demonstrated sensitivity of 64% (95% CI: 53%–74%), without significant improvement in sensitivity as the number of multidetector arrays increased. The issue of insufficient sensitivity is highlighted in Figures 2, 3, a case in which a patient with a negative CTA underwent DSA due to high suspicion for BCVI, and was found to have a vertebral artery dissection.

Another concern regarding CTA screening of BCVI relates to its relatively low positive predictive value. For example, Grandhi et al. (19) reported a positive predictive value as low as 30% in low-grade injuries. This highlights an important conundrum when considering treatment of patients with BCVI. For low-grade injuries, patients are either started on antiplatelet therapy or they are monitored and reimaged after a short interval. Patients with higher-grade injuries often undergo further advanced imaging such as DSA and possible intervention or anticoagulation. The preference at our institution is to administer aspirin immediately to all patients who present with BCVI; however, in situations such as polytrauma with cardiopulmonary instability, hollow organ injury, or patients who present with severe TBI requiring operative intervention, a multidisciplinary discussion is held to discuss antithrombotic strategy (aspirin vs. heparin) and timing, as consideration is given to injury grade, concomitant injuries, and stability of the patient. This is particularly prescient given the increased requirement for blood transfusions in patients started on aspirin for treatment of BCVI (20). Thus, the low positive predictive value can have a notably detrimental effect on patients that are misdiagnosed.

In spite of its shortcomings, CTA is a mainstay in the diagnosis and treatment of patients with BCVI. When screening modalities were compared by Kaye et al. (21) in 2011, CTA screening was associated with the lowest stroke rates and lowest overall cost as well. CTA has also significantly decreased delay in diagnosis of BCVI, as indicated by Eastman et al. (5) who found that time to diagnosis decreased from 31 to 2.6 h after transitioning from DSA to CTA for screening. CTA also allows for a cost-effective and meaningful avenue for short interval re-imaging, which is particularly useful in lower-grade injuries (22). Although repeat imaging is routinely conducted with CTA for these reasons, it does incur increased radiation doses for patients over time, which is especially compounded given that patients with polytrauma often undergo many scans for other injuries. The possibility of imaging BCVIs with non-irradiating imaging studies, such as MRI, would alleviate this issue and reduce cumulative radiation exposure for this patient cohort.

**Figure 4:**
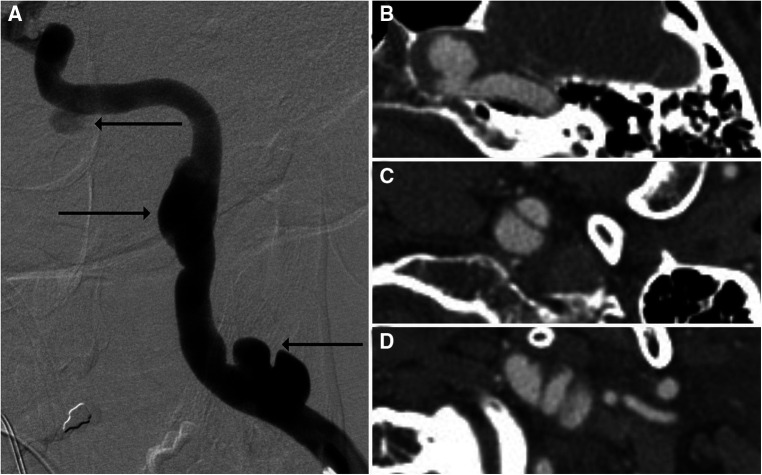
Imaging of a 52-year-old man who presented with headache. DSA (**A**) and CTA (**B–D**) showed multiple pseudoaneurysms related to previous dissection involving the left cervical and petrous internal carotid artery segments, with arrows on DSA corresponding to CTA slices.

It is also crucial to note that the quality of modern CTAs is such that many distinguishing characteristics are readily observable, in turn lending reliability to grading classifications. For example, Grade I injuries typically present as intimal tears causing luminal irregularities, which are minor and can be mistaken for other conditions such as vasospasm. Should the intimal tear progress, it may result in a raised intimal flap, which appears as a linear filling defect, and should this result in 25%–50% luminal stenosis, the injury is then classified as Grade II. Grade III injuries consist of pseudoaneurysms (Figure 4), Grade IV injuries entail occlusion, and Grade V injuries consist of transections with active extravasation (23). Certain high-risk radiographic factors may also be identifiable *via* CTA. For example, Lauerman et al. (24) retrospectively reviewed a series of 312 BCVIs and found that grade III carotid BCVIs (pseudoaneurysms) presenting with luminal stenosis underwent higher rates of endovascular intervention compared with those without luminal stenosis.

Other CT-related imaging studies are still being examined, such as whole-body CT (WBCT) and dual-energy CT. WBCT can be rapidly obtained and is easily conducted in patients who have limited time for studies because of other acute injuries (25). WBCT generally consists of a head CT followed by 40- or 64-multidetector CT with contrast of the rest of the neck, chest, abdomen, and pelvis. This may allow for rapid detection of BCVIs, followed by further studies such as CTA at a later time when more time can be allotted to specialty studies. Laser et al. (25) demonstrated concordance in injury assessment in 48% of 319 cases in which WBCT and CTA were both performed. Dual-energy CT consists of concomitant acquisition of two sets of images *via* two different x-ray spectra. This allows for more efficient bone subtraction, eliminating the need for a separate scan and decreasing radiation exposure (26). This may also yield clearer delineation of pathologies lying near the skull base, allowing for better characterization of these lesions (27). Clearly, advancements in CT technology are likely to further influence BCVI management in the future.

### Magnetic resonance imaging modalities

Although magnetic resonance (MR) has generally been excluded as a screening tool because of high cost and length of study time, considerable effort has still been dedicated to assessing its utility in BCVI characterization. MR angiography (MRA) has the benefit over CTA that it avoids ionizing radiation to the patient and can simultaneously assess for soft tissue injury such as strokes. MRA is an intuitive choice given its orientation toward vascular characterization; however, studies have not demonstrated significantly better results with MRA compared with CTA. For example, Biffl et al. (28) demonstrated sensitivity in the range of 75%, with specificity of only 67% and positive predictive value of 43% with MRA. MR imaging may also aid in the characterization of high-risk features in BCVIs. For example, Wu et al. (29) found that intramural hematomas, irregular vessel surface, intraluminal thrombus, and severe stenosis were more common in patients who sustained strokes. Upon conducting multivariate analysis, they identified a significant association between irregular vessel surface and the presence of intraluminal thrombus with acute ischemic stroke (odds ratio 4.29 (95% CI 1.61–11.46, *P* = 0.004) and 7.48 (95% CI 1.64–34.07, *P* = 0.009), respectively).

**Figure 5:**
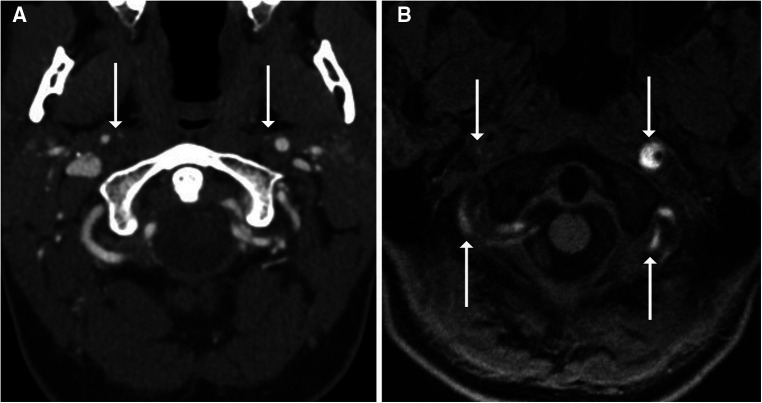
Imaging of a 28-year-old woman who presented with neck pain and headache immediately after delivering her first child. CTA (**A**) showed narrowing of the right more than left cervical internal carotid arteries with suspected crescentic intramural hematomas (arrows). MRA was performed with MPRAGE (**B**), which confirmed bilateral internal carotid artery and vertebral artery intramural hematomas, with many areas showing crescentic T1 hyperintense signal (arrows).

It is generally thought that the rapid movement caused by intraluminal blood flow causes too much artifact to accurately assess BCVIs *via* some MR modalities; however, sequences such as double-inversion recovery black-blood imaging (DIR-BBI) can negate the signal from flowing blood, making them an ideal choice for assessing vessels in BCVI (30). Hunter et al. (30) compared CTA and MRI DIR-BBI in patients with BCVI and found no significant difference in number of dissections detected. A significantly greater number of intimal flaps and wall thickening consistent with intramural hematoma were also identified on MRI DIR-BBI when compared with CTA. MRI with vessel wall imaging (VWI) has also been compared with CTA in the assessment of BCVIs. In a study by Vranic et al. (31), six patients with BCVIs identified on CTA underwent MRI-VWI, with multiple neuroradiologists assessing the images; the results demonstrated significant concordance among neuroradiologists on identifying and grading BCVIs *via* MRI-VWI. Vessel wall enhancement on MRI-VWI may also aid in identifying areas of injury beyond the discrete borders of a dissection flap, indicative of a greater degree of injury than may have been described with non-contrast imaging (32). Conventional T1-weighted fat saturation techniques have recently been replaced with heavily T1-weighted sequences such as MPRAGE (magnetization prepared rapid acquisition echo), given higher interrater reliability for intramural hematoma detection and its independent association with acute territorial infarction (Figure 5) (33). Other sequences such as three-dimensional simultaneous non-contrast angiography and intra-plaque hemorrhage (3D-SNAP) have been assessed in their utility for describing BCVIs as well. Among other MR imaging sequences including 3D-time of flight, T2, 3D-proton density, 3D-T1-volume isotropic turbo-spin echo, 3D-SNAP, and 3D-T1 contrast-enhanced sequences, 3D-SNAP was the most sensitive in detecting intramural hematomas (34). Adam et al. (35) assessed the utility of diffusion-weighted imaging (DWI) in assessing intramural hematomas. In this study, a senior and junior radiologist evaluated the same series of 50 patients with DWI and demonstrated a sensitivity of 86% and 90%, respectively, for detecting intramural hematomas.

Another appealing aspect of MRI is its potential to characterize the acuity of vessel injuries. Patients frequently present with subtle findings concerning for a possible BCVI, and in the context of blunt trauma, it is often extremely difficult to differentiate non-traumatic anatomic variants from clinically significant lesions. Hunter et al. (30) found that patients with BCVI demonstrated periadventitial T2 hyperintensity, a finding the authors attributed to acute inflammatory edema that would not be identified without MRI. In addition, as in extravascular intracranial hematomas, the intensity of blood in intramural hematomas changes over time, meaning that hematoma intensity can be used as a marker of chronicity (36). Another sequence modifier that may aid in the differentiation of true BCVIs from non-acute lesions is the volume isotropic turbo-spin-echo acquisition (VISTA) sequence. In a study by Sakurai et al. (37), the VISTA sequence was shown to aid in the differentiation of intramural hematomas from atherosclerotic plaques and intraluminal thrombi in cases of both carotid and vertebral artery dissections. Overall, while MRI/MRA on its own may not produce a sufficiently compelling reason for its use in BCVI, recent advances in MR technology have produced sequences that exquisitely describe the vessel characteristics and may aid in demonstrating high-risk features of injuries that help guide clinical management.

### Transcranial doppler ultrasound

Transcranial doppler ultrasound (TCD) has been available and evaluated for use in BCVI for several decades. The appeal of TCD is that it is very convenient, easy to obtain without patient travel, and demands a very low cost to use. Unfortunately, it has not demonstrated consistent ability to correctly identify BCVIs, with Mutze et al. (38) reporting a sensitivity of 38.5% (95% CI: 13.9%, 68.4%). The development of TCD with emboli detection, however, has largely altered the level of utility for TCD in BCVI, particularly in high-grade BCVIs. Morton et al. (39) described a series of 59 grade IV BCVIs, of which 11 affected the internal carotid arteries and the remaining 48 affected the vertebral arteries. All patients in the internal carotid artery group who subsequently had strokes demonstrated ≥8 microemboli per hour on TCD with emboli detection. Patients with vertebral artery injuries that developed strokes, by contrast, did not demonstrate microemboli on TCD with emboli detection (37). Another study conducted by Bonow et al. (40) used assessment with TCD with emboli detection in 1,146 patients with BCVI. The development of delayed stroke (>24 h after arrival) was associated with at least one microembolus detected on TCD with emboli detection (risk ratio 5.05; 95% CI 1.41–18.13). The likelihood of stroke development also increased with the number of microemboli detected, as well as persistence of emboli over the course of multiple days. Again, there was no association between positive TCD with emboli detection studies and strokes in posterior circulation after vertebral artery injury. TCD with emboli detection may therefore be a useful diagnostic tool in stratifying risk for stroke after BCVI in cases of internal carotid artery but not necessarily vertebral artery injury.

## Conclusion

Although often clinically subtle or asymptomatic, BCVI is a very common and potentially dangerous injury in patients that have experienced blunt force traumas. Although traditional catheter-based cerebral angiography is still the gold standard for diagnosis, significant advancements in CT, MR, and TCD technology have allowed for more reliable identification of BCVIs and improved risk stratification of these injuries to aid in clinical management. As practices currently stand, patients with suspected BCVIs are screened with CTA, with escalation to other studies being reserved for higher-grade injuries (often prompting formal angiography) or higher risk factors such as the presence of pseudoaneurysms/intraluminal thrombi/intramural hematoma (cases that may benefit from TCD emboli detection and MRI). Further studies are still required to replicate the results of these newer technologies on a larger scale to fully determine their niche in the diagnosis and management of BCVIs.
